# Intensivpflegerische Versorgung von Patient:innen mit [infarktbedingtem], kardiogenen Schock: Kurzversion der S1-Leitlinie

**DOI:** 10.1007/s00063-022-00945-1

**Published:** 2022-08-30

**Authors:** C. Hermes, T. Ochmann, Deutsche Gesellschaft für Internistische Intensivmedizin und Notfallmedizin e. V. (DGIIN), C. Keienburg, Deutsche Gesellschaft für Fachkrankenpflege und Funktionsdienste e. V. (DGF), M. Kegel, Deutsche Gesellschaft Interdisziplinäre Notfall- und Akutmedizin e. V. (DGINA), D. Schindele, Deutsche Interdisziplinäre Vereinigung für Intensiv- und Notfallmedizin e. V. (DIVI), J. Klausmeier, Deutsche Gesellschaft für Kardiologie – Herz- und Kreislaufforschung e. V. (DGK), E. Adrigan, Österreichische Gesellschaft für Internistische und Allgemeine Intensivmedizin und Notfallmedizin e. V. (ÖGIAIN)

**Affiliations:** 1 Hochschule für Angewandte Wissenschaften Hamburg (HAW Hamburg), Alexanderstraße 1, 20099 Hamburg, Deutschland; 2 Klinik für Kardiologie, Angiologie und Internistische Intensivmedizin, kath. Marienkrankenhaus Hamburg, Hamburg, Deutschland; 3 Zentrum für Kardiologie, Universitätsmedizin Mainz, Langenbeckstr. 1, 55131 Mainz, Deutschland; 4 Klinikverbund Bremen, Bildungsakademie der Gesundheit Nord gGmbH, Bremen, Deutschland; 5 RKH Akademie, Klinikum Ludwigsburg-Bietigheim, Regionale Kliniken Holding RKH GmbH, Ludwigsburg, Deutschland; 6 Contilia Institut für Pflege- und Gesundheitsberufe, St. Marien-Hospital Mülheim an der Ruhr, Mülheim an der Ruhr, Deutschland; 7 Abteilung für allgemeine und internistische Intensivmedizin, Universitätsklinikum Innsbruck, Innsbruck, Österreich

**Keywords:** Advanced Nursing Practise, Qualitätsmanagement, Pflegewissenschaft, Fachkrankenpflege, Pflichtfortbildung, Advanced nursing practise, Quality management, Nursing science, Critical care nurse, Mandatory training

## Abstract

**Hintergrund:**

Herz-Kreislauf-Erkrankungen und der (infarktbedingte) kardiogene Schock zählen zu den häufigsten Todesursachen in Deutschland. Eine adäquate klinische Versorgung stellt die Krankenhäuser oft vor große Herausforderungen. Die komplexe Versorgung der Patient:innen im multiprofessionellen Team stellt hohe Anforderungen an alle am Versorgungsprozess Beteiligten. Da besonders die Pflegefachpersonen im engen Patient:innenkontakt stehen und die Therapie maßgeblich mitgestalten und steuern, ist eine nationale, (intensiv)pflegerische Leitlinie dringend erforderlich.

**Methoden:**

Im Rahmen des Leitlinienprogramms der Arbeitsgemeinschaft der Wissenschaftlichen Medizinischen Fachgesellschaften e. V. (AWMF) wurde unter Beteiligung von sechs Fachgesellschaften eine S1-Leitlinie entwickelt und im Mai 2022 veröffentlicht. Die Leitliniengruppe legte relevante Themengebiete fest, die durch eine systematische Literaturrecherche in Peer-Review-Journalen bearbeitet wurden. Aufgrund der S1-Klassifikation wurde keine gesonderte Evidenzaufbereitung vorgenommen.  Zur Einstufung der Empfehlungen wurde ein formaler Konsensbildungsprozess durchgeführt.

**Ergebnisse:**

Die Leitlinie enthält 36 Empfehlungen, die sich von der pflegerischen Versorgung in der Zentralen Notaufnahme über das Herzkatheterlabor und die Intensivstation bis zur Nachsorge erstrecken. Zudem werden Empfehlungen zu notwendigen Qualifikationen und strukturellen Voraussetzungen in den jeweiligen Bereichen getroffen, um einen qualitativ hochwertigen (pflegerischen) Versorgungsprozess zu gewährleisten.

**Fazit:**

Dies ist die erste nationale intensivpflegerische Leitlinie. Sie richtet sich an Pflegefachpersonen, die in die Versorgung von Patient:innen mit (infarktbedingtem) kardiogenen Schock involviert sind. Die Leitlinie ist bis zum 30.12.2026 gültig.

**Zusatzmaterial online:**

Die Online-Version dieses Beitrags (10.1007/s00063-022-00945-1) enthält weitere Abbildungen und Checklisten.

## Abstract (en)

**Background:**

Cardiovascular diseases and (infarct-related) cardiogenic shock are among the most frequent causes of death in Germany. Adequate clinical care often poses great challenges for hospitals. The complex care of patients in a multi-professional team places high demands on all those involved in the care process. Since nurses in particular are in close contact with patients and play a decisive role in shaping and controlling therapy, a national (intensive) care guideline is urgently needed.

**Methods:**

Within the framework of the guideline programme of the Association of the Scientific Medical Societies in Germany (AWMF), an S1 guideline was developed with the participation of six professional societies and published in May 2022. The guideline group defined relevant topics, which were processed through a systematic literature search in peer-reviewed journals. Based on the S1 classification, no separate evidence review was conducted. A formal consensus-building process was used to classify the recommendations.

**Results:**

The guideline contains 36 recommendations ranging from nursing care in the central emergency department to the cardiac catheterisation laboratory, intensive care unit and follow-up care. In addition, recommendations are made on the necessary qualifications and structural requirements in the respective areas in order to ensure a high-quality (nursing) care process.

**Conclusion:**

This is the first national intensive care guideline. It is aimed at nurses involved in the care of patients with (infarct-related) cardiogenic shock. The guideline is valid until 30.12.2026.

## Hintergrund zur Leitlinie

Leitlinien der Arbeitsgemeinschaft der Wissenschaftlichen Medizinischen Fachgesellschaften (AWMF) sind systematisch entwickelte Hilfen, die in erster Linie dem ärztlichen Dienst zur Entscheidungsfindung in spezifischen Situationen dienen. Obgleich an der Erstellung und Umsetzung verschiedene Berufsgruppen und Fachgesellschaften beteiligt sind, ist insbesondere auf den Intensivstationen und in den angrenzenden Bereichen der Akut- und Notfallmedizin für die Leitlinienumsetzung ein enger interprofessioneller und interdisziplinärer Austausch sowie kollegiale Zusammenarbeit essenziell. In zahlreichen Leitlinien finden sich wichtige Impulse und Hinweise aus den Gesundheitsfachberufen außerhalb der Medizin. Auch bei der Erstellung der S3-Leitlinie „Infarktbedingter kardiogener Schock – Diagnose, Monitoring und Therapie“ waren ursprünglich Kolleg:innen der Pflege beteiligt. Aus verschieden Umständen heraus musste im weiteren Verlauf der Novellierung dieser S3-Leitlinie auf die pflegerische Expertise verzichtet werden. Insbesondere Prof. Dr. Karl Werdan war es immer ein besonderes Anliegen, diese pflegerische Expertise nicht verlorengehen zu lassen. Dieser Umstand hat die Sektion Pflege der Deutschen Gesellschaft für internistische Intensivmedizin und Notfallmedizin (DGIIN) mit einstimmiger Zustimmung des Vorstands der DGIIN veranlasst, eine pflegerische S1-Leitline als Ergänzung zur bestehenden S3-Leitlinie zur Versorgung von Patient:innen mit (infarktbedingtem) kardiogenem Schock auf den Weg zu bringen. Die Bemühungen wurden aktiv durch die pflegerischen Kolleg:innen der DGIIN und durch eine engagierte Mitarbeit der beteiligten Fachgesellschaften sowie den neu gegründeten Arbeitsgruppen unterstützt. Dadurch wurde der Weg für diese erstmalig originär intensivpflegerisch initiierte und geleitete Leitlinie geschaffen, die es sich zum Ziel setzt, wichtige pflegerische Aspekte in der fachpraktischen Umsetzung zu benennen; denn es ist eine häufige Fragestellung in der Praxis: Wie sollen die (medizinischen) Empfehlungen einer Leitlinie in der Praxis durch die dafür zuständigen Gesundheitsfachberufe umgesetzt werden.

## Einleitung

Die Versorgung von Patient:innen mit Herz-Kreislauf-Erkrankungen stellt eine große Herausforderung für die deutschen Krankenhäuser dar. Laut Statistischem Bundesamt sind im Jahr 2019 rund 330.000 Menschen an einer Herz-Kreislauf-Erkrankung, 44.000 davon an einem Myokardinfarkt, verstorben. Damit bilden diese Erkrankungen die häufigste Todesursache in Deutschland [32]. Der (infarktbedingte) kardiogene Schock (IKS) ist ein häufiges Krankheitsbild auf deutschen Intensivstationen und geht mit einer hohen Letalität einher. Die intensivpflegerische und intensivmedizinische Versorgung erfordert ein interprofessionelles und interdisziplinäres Team mit standardisierten Abläufen, um u. a. eine frühzeitige koronare Rekanalisation zu erreichen, mit dem Ziel, die Mortalität möglichst zu senken.

Die klinische Versorgung von Patient:innen mit (infarktbedingtem) kardiogenen Schock ist komplex und beginnt bereits mit der Aufnahme in der Zentralen Notaufnahme (ZNA). Der Versorgungsprozess erstreckt sich über das Herzkatheterlabor bis hin zur Intensivstation oder Chest Pain Unit. Bei der Versorgung von Patient:innen mit (infarktbedingtem) kardiogenen Schock kann es jederzeit zu akuten Komplikationen kommen. Daher ist die Einleitung der Sofortmaßnahmen ebenso wichtig wie die Kenntnis der Pflegediagnostik, zutreffender Pflegediagnosen sowie das Vorgehen bei der Nachsorge und Rehabilitation.

Diese S1-Leitlinie soll die Komplexität der pflegerischen Versorgung des IKS, ergänzend zur S3-Leitlinie „Infarktbedingter kardiogener Schock – Diagnose, Monitoring und Therapie“ [34], abbilden und als eine Empfehlung zur Qualitätssicherung und Entscheidungshilfe bei der Versorgung dieser Patient:innengruppe dienen. Neben den bereichsübergreifenden Tätigkeiten benötigen die professionell Pflegenden in den einzelnen Abteilungen zusätzlich spezifische Kompetenzen zur Versorgung der Patient:innen vor Ort. In der vorliegenden Leitlinie sollen sowohl die bereichsübergreifenden Aufgaben als auch die unterschiedlichen Qualifizierungen berücksichtigt werden.

Die Qualifizierungsmöglichkeiten für professionell Pflegende in Deutschland sind derzeit vielfältig, und die Implementierung der einzelnen Berufsabschlüsse in der Praxis ist sehr heterogen. Die Steuerungsgruppe dieser Leitlinie hat sich auf folgende Unterscheidungen in der Qualifizierung der Pflegenden geeinigt:

- Pflegefachpersonen bzw. Pflegefachkraft bezeichnet Pflegende mit einer 3‑jährigen Ausbildung oder primär qualifizierendem Studium mit Erlaubnis zur Führung der Berufsbezeichnung gemäß § 1 nach Pflegeberufegesetz (PflBG).
- Weitergebildete Pflegefachpersonen bzw. Pflegefachkraft bezeichnet Pflegende, welche nach der 3‑jährigen Ausbildung zusätzlich eine staatlich anerkannte bzw. nach DKG-Empfehlungen durchgeführte 2‑jährige berufsbegleitende Weiterbildung zur Intensivpflege und Anästhesie oder Notfallpflege oder ein gleichwertiges Studium absolviert haben.
- Spezialisierte Pflegefachpersonen bzw. Pflegefachkraft bezeichnet Pflegende, welche nach der 3‑jährigen Ausbildung eine Zusatzqualifikation in Form einer zertifizierten, fachspezifischen Fortbildung, z. B. Pflegeexpert:in (Chest Pain Unit) absolviert haben.

Die Leitliniengruppe stimmt darin überein, dass eine akademische Qualifikation im Sinne einer Advanced Nursing Practice (ANP) auf Masterniveau von o. g. Pflegefachpersonen eine sinnvolle Ergänzung sein kann [9]. ANPs sollen entsprechend der internationalen Definition des ICN qualifiziert sein und über eine fachpraktische Spezialisierung im jeweiligen Fachbereich, in dem sie eingesetzt werden, verfügen.

## Methode

Federführende und unabhängig finanzierende Fachgesellschaft ist die Deutsche Gesellschaft für Internistische Intensivmedizin und Notfallmedizin e. V. (DGIIN). Bei der Anmeldung der Leitlinie war die Beteiligung folgender Fachgesellschaften vorgesehen:

- Deutsche Gesellschaft für Internistische Intensivmedizin und Notfallmedizin e. V. (DGIIN)
- Deutsche Gesellschaft Interdisziplinäre Notfall- und Akutmedizin e. V. (DGINA)
- Deutsche Gesellschaft für Kardiologie – Herz- und Kreislaufforschung e. V. (DGK)
- Deutsche Interdisziplinäre Vereinigung für Intensiv- und Notfallmedizin e. V. (DIVI)
- Deutsche Gesellschaft für Fachkrankenpflege und Funktionsdienste e. V. (DGF)
- Österreichische Gesellschaft für Internistische und Allgemeine Intensivmedizin und Notfallmedizin e. V. (ÖGIAIN).

Alle Fachgesellschaften waren durch Mandatsträger vertreten und haben (nicht stimmberechtigte) Arbeitsgruppen gebildet. Die Leitlinienentwicklung wurde von Dr. Monika Nothacker, MPH (AWMF), Berlin, und Prof. Dr. Karl Werdan, Halle, methodisch begleitet. Da es sich um eine S1-Leitlinie handelt, wurde keine gesonderte Evidenzaufbereitung vorgenommen.

Eine evidenzbasierte klinische Entscheidungsfindung ist zentraler Bestandteil der täglichen Praxis von Pflegefachkräften auf einer Intensivstation und in anderen Funktionsbereichen, wie z. B. in der Notaufnahme oder auf einer Chest Pain Unit (CPU), und basiert auf einer analytischen Beurteilung von klinischen und apparativ erhobenen Messwerten [31]. Klinische Entscheidungsfindung ist ein wesentlicher Bestandteil professioneller Pflege. Pflegende haben den häufigsten Patient:innenkontakt und stellen somit das bindende Glied im interprofessionellen Team dar. Es obliegt den Pflegefachkräften, Veränderungen des Patient:innenzustands zu erkennen und auf der Grundlage ihrer klinischen Expertise geeignete Maßnahmen im interprofessionellen Team einzuleiten [25].

In dieser S1-Leitlinie werden essenzielle Aspekte mit dem Fokus auf die pflegerische Versorgung aufgezeigt. Die täglichen Herausforderungen der professionell Pflegenden, die akut lebensbedrohlichen Zustände der Patient:innen zu erkennen und zu behandeln, erfordern ein hohes Maß an Fähigkeiten und Fertigkeiten, die mit Erfahrung im Beruf und ständiger Fort- und Weiterbildung gefestigt werden müssen.

Die Leitliniengruppe legte bei ihrer konstitutionellen Sitzung 10 generelle Themengebiete und Überschriften fest, welche in der Leitlinie durch eine systematische Literaturrecherche in Peer-Review-Journalen bearbeitet wurden. Nur in Ausnahmefällen wurde auf „graue Literatur“ oder andere Medien zurückgegriffen. Im vorliegenden Dokument handelt es sich um die Kurzversion der Leitlinie mit ausgesuchten Empfehlungen. Eine ausführliche Langversion ist veröffentlicht [18]. Unter https://www.awmf.org/leitlinien/detail/ll/113-002.html sind Interessenkonflikte, Evidenzberichte und Leitlinienberichte abrufbar. Das methodische Vorgehen richtete sich nach dem AWMF-Regelwerk (http://www.awmf-leitlinien.de). Die Formulierung von Empfehlungen erfolgte in 3 Graden: starke Empfehlung (soll/soll nicht), Empfehlung (sollte/sollte nicht), offene Empfehlung (kann/kann verzichtet werden). Empfehlungen wurden im Expertenkonsens (Tab. 1) formuliert. Dabei wurden die durch die Recherche gefundenen, geeigneten Studien in die Diskussion mit eingebracht. Interessenkonflikte mit direktem Einfluss auf die Thematik und/oder welche, die zum Ausschluss bei der Abstimmung geführt hätten, wurde bei keinem Leitgruppenmitglied festgestellt. Die Interessenkonflikte und Konsequenzen daraus sind ebenfalls auf der Seite der AWMF einsehbar.

**Table 1:** 

*Starker Konsens*	Zustimmung von mindestens 6 der 7 stimmberechtigten Mitglieder
*Mehrheitlicher Konsens*	Zustimmung von mindestens 5 der 7 stimmberechtigten Mitglieder
*Mehrheitliche Zustimmung*	Zustimmung von mindestens 4 der 7 stimmberechtigten Mitglieder
*Kein Konsens*	Zustimmung von weniger als 4 der 7 stimmberechtigten Mitglieder

### Empfehlungen

Die folgenden Empfehlungen und Hintergrundtexte stellen einen Auszug dar, die gesamte Leitlinie ist hier abrufbar: https://www.awmf.org/uploads/tx_szleitlinien/113-002l_S1_Intensivpflegerische-Versorgung-PatientenInnen-mit-Infarktbedingten-kardiogenen-Schock_2022-05.pdf.

### Sofortmaßnahmen

Im Mittelpunkt der Sofortmaßnahmen beim (infarktbedingten) kardiogenen Schock steht die Symptomlinderung und Stabilisierung der hämodynamischen Situation. Als erweiterte Kompetenzen im Bereich der Notfallpflege, Intensivpflege oder CPU lässt sich hier insbesondere die umfassende und engmaschige Einschätzung und Überwachung des Gesundheitszustands in der komplexen Versorgung von Patient:innen mit IKS ableiten. Ein besonderer Fokus liegt auf der Beurteilung der Herz-Kreislauf-Situation, ggf. durch ein erweitertes Monitoring, sowie die Überwachung weiterer Organfunktionen, wie z. B. Nierenfunktion, Lungenfunktion oder Bewusstseinslage.

#### Empfehlung 2

- Zur Erleichterung der Sauerstoffversorgung *sollen* Patient:innen im kardiogenen Schock bei RR-Werten über 100 mm Hg in 20–45° Oberkörperhochlagerung, idealerweise als Herzbettlagerung, gebracht werden.
- Pflegefachpersonen *sollen* die Sauerstoffversorgung im Organismus durch zusätzliche Applikation von Sauerstoff, unter Berücksichtigung der S3-Leitlinie zur Sauerstofftherapie, über einen situationsgerechten Applikationsweg sicherstellen.
- Die Gabe von Medikamenten und Infusionslösungen *soll* in der Akutsituation durch die Anlage mehrerer peripher-venöser Zugänge sichergestellt werden. Gelingt dies nicht, *sollte* die Anlage eines intraossären Zugangs veranlasst und alle Materialien dafür vorbereitet werden.
- Pflegefachpersonen *sollen* ärztlich angeordnete Schmerztherapie einleiten und fortführen, bis eine klare Symptombesserung vorliegt.
- Auf der Grundlage qualifizierter Fachkenntnisse und Erfahrung *sollen* weitergebildete Pflegefachpersonen die kontinuierliche Herz-Kreislauf-Überwachung der Patient:innen übernehmen. Anhand des kontinuierlichen Basismonitorings *sollen* pathologische Herzrhythmusstörungen, gefährliche Hypotonien und niedrige SpO_2_ sicher erkannt werden.
- Bei pathologischen Veränderungen *soll* eine zeitnahe Informationsweitergabe an den zuständigen ärztlichen Dienst erfolgen.
- Die Versorgung von Patient:innen im kardiogenen Schock *soll* in ständiger Reanimationsbereitschaft erfolgen.

**Starker Konsens – GoR ↑↑↑**.

Konsentiert: 7/7 der Delegierten.

### Vorbehaltsaufgaben

Insbesondere Pflegefachpersonen nach abgeschlossener 3‑jähriger pflegerischer Grundausbildung und idealerweise auch mit einer zusätzlichen (Fach‑)Weiterbildung sind in den intensiv- und notfallpflegerischen Versorgungsprozess integriert. Sie übernehmen pflegerische Vorbehaltsaufgaben im Rahmen des Pflegeprozesses und führen Aufgaben entsprechend ihrer erworbenen Fähigkeiten und Fertigkeiten selbstständig aus. Dazu gehört, neben den Schritten des Pflegeprozesses und Einschätzung der Pflegediagnosen, auch die selbstständige Einleitung lebenserhaltender Sofortmaßnahmen sowie die eigenständige Durchführung ärztlich angeordneter Maßnahmen der medizinischen Diagnostik, Therapie oder Rehabilitation [28]. Im Rahmen des pflegediagnostischen Prozesses erfolgt die Identifikation spezifischer Pflegediagnosen. Durch eine entsprechende Zusatzqualifikation und/oder Spezialisierung in den Fachbereichen der Intensivpflege, Notfallpflege oder CPU erschließt sich ein erweitertes Handlungsfeld der Pflegefachpersonen im Rahmen des intensivpflegerischen Versorgungsprozesses und der Notfallversorgung von Patient:innen mit IKS [7]. Ihre Schlüsselfunktion im kontinuierlichen Patient:innenkontakt garantiert eine lückenlose Versorgungsqualität im interprofessionellen Versorgungsprozess [19].

#### Empfehlung 3

- Pflegefachpersonen *sollen* die Schritte des Pflegeprozesses als Vorbehaltsaufgabe der pflegerischen Profession selbstständig und zielorientiert ausführen.
- Pflegefachpersonen *sollen* die passenden Pflegediagnosen anhand der jeweiligen Definition innerhalb von 24 h nach Aufnahme identifizieren und die Diagnosestellung mithilfe der bestimmenden Merkmale oder Kennzeichen einmal täglich überprüfen und entsprechend dokumentieren.
- Pflegefachpersonen *sollen* die ermittelten Pflegediagnosen priorisieren und die daraus resultierenden Maßnahmen gezielt in den intensivpflegerischen Versorgungsprozess einbringen.
- Pflegefachpersonen *sollen* im Notfall lebenserhaltende Sofortmaßnahmen einleiten und bis zum Eintreffen des ärztlichen Dienstes selbstständig durchführen.
- Pflegefachpersonen *sollen* ärztlich angeordnete Maßnahmen der medizinischen Diagnostik, Therapie und Rehabilitation eigenständig durchführen.
- Pflegefachpersonen *sollen *geeignete Assessmentinstrumente zur Einschätzung der Patient:innensituation adäquat und sicher anwenden und interpretieren sowie passende Handlungen daraus ableiten können.

**Starker Konsens – GoR ↑↑↑**.

Konsentiert: 7/7 der Delegierten.

(Weitergebildete) Pflegefachpersonen übernehmen die Betreuung der Patient:innen und deren Angehörigen in einer existenziellen Notlage. Sie zeigen einen professionellen Umgang mit den akuten Ängsten der Patient:innen und ihrer Angehörigen unter Berücksichtigung der aktuellen Lebenssituation. Falls erforderlich, stellen sie umfassend Lebensaktivitäten und Grundbedürfnisse der Patient:innen unter Berücksichtigung des aktuellen Gesundheitszustands sicher.

Eine auf die 3‑jährige pflegerische Grundausbildung aufbauende Fachweiterbildung zur Fachpflegekraft (z. B. Notfallpflege, Intensivpflege und Anästhesie) stellt einen elementaren Baustein in der Weiterentwicklung der Wissensbasis und der Kompetenzen von Pflegefachkräften dar.

#### Empfehlung 4

- Im direkten Patient:innenkontakt *sollen* weitergebildete bzw. spezialisierte Pflegefachpersonen die Herz-Kreislauf-Situation und weitere Organfunktionen (wie z. B. Nierenfunktion, Lungenfunktion oder Bewusstseinslage) überwachen und beurteilen.
- Neben der Durchführung ärztlich angeordneter Diagnostik und Therapie *sollen* weitergebildete Pflegefachpersonen diese auch überwachen und situationsgerecht steuern (z. B. Katecholamintherapie, Beatmungstherapie, Ernährungstherapie, Sedierungs- und Schmerzmanagement).
- Fachpflegende *sollen* zur Einschätzung auch das erweiterte hämodynamische Monitoring interpretieren und beurteilen können.
- Durch zusätzlich im Rahmen einer berufsbegleitenden Weiterbildung erworbenes Wissen, Fertigkeiten und Sozialkompetenz *können* fachweitergebildete Pflegefachpersonen evidenzbasierte, steuernde, klinisch einschätzende und therapieführende Aufgaben in der komplexen intensivpflegerischen bzw. notfallpflegerischen Versorgungssituation eigenverantwortlich und selbstständig übernehmen.

**Starker Konsens – GoR ↑↑↑**.

Konsentiert: 7/7 der Delegierten.

- Pflegefachkräfte, die in die Versorgung des IKS eingebunden sind, *sollen* sich mindestens einmal jährlich spezifischen Fortbildungen zum Themengebiet von in Summe nicht weniger als 8 Unterrichtseinheiten (UE) unterziehen.
- Diese jährlichen Fortbildungen *sollen* zusätzlich zu der Reanimations- und Notfallfortbildung sowie den gängigen Pflichtfortbildungen erfolgen.
- Für die Versorgung von Patient:innen mit IKS *sollten* bevorzugt weitergebildete Pflegefachpersonen eingesetzt werden.
- Ist die primäre Versorgung durch weitergebildete Pflegefachpersonen nicht möglich, *soll* in jeder Schicht unmittelbar eine entsprechende Expertise für die eingeteilten Kolleg:innen einholbar sein, z. B. durch weitergebildete Pflegefachpersonen als freigestellte Schichtleitung und/oder im Leitungsdienst der Station.

**Starker Konsens – GoR ↑↑↑**.

Konsentiert: 7/7 der Delegierten.

### Basismonitoring

Patient:innen mit (infarktbedingtem) kardiogenen Schock sollen ab dem Zeitpunkt der Aufnahme mit einer apparativen, kontinuierlichen Herz-Kreislauf-Überwachung ausgestattet werden.

#### Empfehlung 5

- Die Konfiguration des Monitorings *soll* einem stationseinheitlichen Standard folgen.
- Durch gezielte Interpretation der Klinik und Anamnese *sollen* darüber hinaus Erkenntnisse zu Schmerzdauer, Schmerzcharakter, Schmerzlokalisation sowie spezifischen Begleitsymptomen erhoben werden.

**Starker Konsens – GoR ↑↑↑**.

Konsentiert: 7/7 der Delegierten.

#### Empfehlung 6

- Zu jeder Patient:innenübernahme bzw. zu jedem Schichtbeginn *soll* eine protokollbasierte, standardisierte Antrittskontrolle (Online-Zusatzmaterial Anhang 4 – Checkliste Antrittskontrolle) im Zimmer und am Bett erfolgen.
- Im Rahmen der Antrittskontrolle *sollen* Assessments zu Vigilanz/Agitation, Schmerz und Delir durch die Pflegefachpersonen eigenständig erhoben werden.
- Die Patient:innenübergabe *sollte* sich an einem systematischen Übergabeprotokoll orientieren (z. B. Online-Zusatzmaterial Anhang 3 – Übergabeschema SBAR.)
- Die Patient:innenübergabe *sollte* im 4‑Augen-Prinzip am Bett durchgeführt werden.

**Starker Konsens – GoR ↑↑↑**.

Konsentiert: 7/7 der Delegierten.

### Klinische Untersuchung/Situationseinschätzung

Eine klinische Untersuchung der Patient:innen soll mindestens alle 8 h und bei Veränderung des Patient:innenzustands durchgeführt werden [34]. Ein strukturiertes Vorgehen und die Verwendung von Scoringsystemen helfen den Pflegefachkräften dabei, komplexe Situationen zu erfassen und geeignete Handlungen einzuleiten [6]. Bei kritisch kranken Patient:innen wird eine klinische Untersuchung anhand des ABCDE(F)-Schemas empfohlen. Das ABCDE(F)-Schema besteht aus einem Untersuchungsgang und einem Behandlungsgang [20, 30, 33] und sollte von weitergebildeten Pflegefachkräften eigenständig und selbstständig angewandt werden (siehe Online-Zusatzmaterial Anhang 2 – Flowchart ABCDE(F)-Schema).

#### Empfehlung 7

- Eine strukturierte, umfassende klinische und apparative Patient:inneneinschätzung anhand des ABCDE(F)-Schemas *soll* im Rahmen des täglichen Bettplatzchecks durchgeführt werden.
- Eine Reevaluation anhand des ABCDE(F)-Schemas *soll* bei einer Veränderung des Patient:innenzustands durchgeführt werden.
- Weitergebildete Pflegefachpersonen *sollten* das ABCDE(F)-Schema eigenverantwortlich und selbstständig durchführen und geeignete Erstmaßnahmen im Rahmen der Organisationsvorgaben einleiten.

**Starker Konsens – GoR ↑↑↑**.

Konsentiert: 7/7 der Delegierten.

### ECLS/ECMO

Die ECLS/ECMO-Therapie wird in Deutschland seit vielen Jahrzehnten eingesetzt [11] und stellt auch in der Therapie des kardiogenen Schocks eine Behandlungsmöglichkeit dar – vor allem, wenn sich Patient:innen im (infarktbedingten) kardiogenen Schock nicht zeitnah stabilisieren lassen [34]. Grundsätzlich müssen zwei Bezeichnungen unterschieden werden: „extracorporeal life support“ (ECLS; auch venoarterielle extrakorporale Membranoxygenierung, VA-ECMO, genannt) zur partiellen oder kompletten Übernahme der Herz- und Lungenfunktion und venovenöse extrakorporale Membranoxygenierung (VV-ECMO) als Unterstützungsmaßnahme bei Lungenschäden [21]. Kontrollparameter zur ECLS-Therapie finden Sie Beispielhaft im Online-Zusatzmaterial Anhang 5 – Checkliste ECLS und Anhang 6 – Kontrollparameter ECLS-Therapie in.

#### Empfehlung 13

- Für die Kontrolle der Oxygenierung unter ECLS *soll* die arterielle Blutgasanalyse bei VA-ECMO ausschließlich an der rechten oberen Extremität durchgeführt werden.
- Unter Einsatz der ECLS/ECMO *soll* mindestens alle 8 h und bei Bedarf die periphere Durchblutung und der Beinumfang überprüft werden. Dazu *kann* neben einer Doppleruntersuchung auch ein manuelles Palpieren der Fußpulse zum Einsatz kommen.
- Bei intubierten Patient:innen *soll* eine neurologische Beurteilung mittels Pupillenkontrolle alle 8 h durchgeführt werden.
- Die Blutzuführenden- und abführenden Lifelines der ECLS/ECMO *sollen* direkt am Punktionsort sowie an mindestens einer weiteren Position in der Umgebung der erkranken Person sicher fixiert werden, um keinen Zug auf die Einstichstelle auszuüben.
- Nach jeder Anlage einer ECLS/ECMO *soll* eine ausreichende Polsterung zwischen Hautflächen und den Kathetern sichergestellt werden, um das Risiko eines Dekubitus zu reduzieren.
- Für die Durchführung von Verbandswechseln bei einer ECLS/ECMO *soll* ein hausinterner Standard oder eine SOP, unter Berücksichtigung aktueller Evidenz und den Empfehlungen des RKI, entwickelt und umgesetzt werden.
- Patient:innen und deren Angehörige *sollen* eine der Situation angemessene Aufklärung zu der Therapie mit einer ECLS/ECMO erhalten.
- Das Material zur Anlage von Kanülen zur mechanischen Kreislaufunterstützung *soll* separat gesammelt vorgehalten werden, um eine mechanische Kreislaufunterstützung ohne eine Verzögerung durch Materialsuche gewährleisten zu können.
- Für die routinemäßige Kontrolle eines ECLS/ECMO-Systems *sollte* ein hausinterner Standard oder SOP entwickelt und umgesetzt werden. Darin *sollen* u. a. die Punkte Flushing des Oxygenators, Kontrolle der Alarmgrenzen, Temperatur- und Gerinnungsmanagement berücksichtigt werden.
- Für die Dokumentation der Überwachungswerte des ECLS/ECMO-Systems *sollte* mindestens alle 4–8 h eine Übertragung in die Patient:innendokumentation erfolgen.

**Starker Konsens – GoR ↑↑↑**.

Konsentiert: 7/7 der Delegierten.

#### Therapeutische Hypothermie nach Reanimation

Etwa die Hälfte der IKS-Patient:innen erleidet initial einen Herz-Kreislauf-Stillstand. Als Folge der dadurch ausgelösten pathophysiologischen Vorgänge im Organismus beginnt unmittelbar bei Wiederherstellung des Spontankreislaufs der Reperfusionsschaden. Daher wird in der Postreanimationsphase ein gezieltes „targeted temperature management“ (TTM) für komatöse ROSC-Patient:innen empfohlen. Ziel ist es, eine milde therapeutische Hypothermie von 32–36 °C für mindestens 24 h aufrechtzuerhalten, um das neurologische Outcome der Patient:innen positiv zu beeinflussen. Die Steuerung der Körpertemperatur sollte dabei nach Möglichkeit aktiv und anhand eines gezielten Temperaturmanagements, unabhängig vom initialen Rhythmus und dem Ort des Kreislaufstillstands, erfolgen [26].

##### Empfehlung 14

- In der Postreanimationsphase *soll* ein feedbackgesteuertes Systems zur Erlangung einer milden therapeutischen Hypothermie verwendet werden.
- Eine dauerhafte *manuelle *Kühlung mit Kühlmatten oder der Benetzung der Haut mit alkoholhaltigen Sprays stellt kein kontinuierliches und sicheres Kühlverfahren dar und *soll* daher *nicht* angewendet werden.
- Die kontinuierliche Temperaturmessung *soll* mittels Blasendauerkatheter oder Ösophagussonde erfolgen.
- Es *soll* eine stündliche Kontrolle der Pupillen erfolgen. Form und Reaktion sind dabei zu dokumentieren.
- Eine engmaschige, situationsgerechte Kontrolle der Elektrolyte (v. a. des Kaliums) und Blutzuckerwerte sowie die zeitnahe Korrektur nach ärztlich vorgegebenen Zielwerten *soll* von Fachpflegenden selbstständig übernommen werden.
- Eine engmaschige Überwachung der Patient:innen auf Anzeichen von Blutungen, z. B. bei der Mundpflege, *wird* aufgrund der Gefahr von Gerinnungsstörungen *empfohlen*.
- Es *soll* eine engmaschige Kontrolle des Hautzustands erfolgen, da durch eine Hypothermie die Mikrozirkulation gestört ist. Zudem *sollten* bei auftretenden Arrhythmien und/oder einer zunehmenden hämodynamischen Instabilität im Rahmen einer Hypothermiebehandlung Mikrolagerungen bevorzugt angewandt und gefährdete Körperstellen mit geeignetem Material unterpolstert werden.
- Im Rahmen der Hypothermiebehandlung *sollten* Manipulationen an Endotrachealtubus und/oder Magensonde minimiert werden, da hierdurch Bradykardien oder kritische Arrhythmien ausgelöst werden können.
- Eine Kontrolle der Diurese, auch um eine Hypovolämie zu vermeiden, *sollte* mittels Stundenurometer nach Bilanzvorgaben erfolgen.
- Zur Senkung oder Vermeidung von Fieber in den ersten 72 h *kann* zu allgemein bekannten physikalischen und medikamentösen Maßnahmen gegriffen werden.
- Zur Vermeidung von Kältezittern *können* Maßnahmen zum Counterwarming, bspw. Wärmedecken oder Handschuhe, erwogen werden.

**Starker Konsens – GoR ↑↑↑**.

Konsentiert: 7/7 der Delegierten.

## Pflegerische Versorgung des (infarktbedingten) kardiogenen Schocks in der zentralen Notaufnahme (ZNA)

Die adäquate Versorgung von Patient:innen mit IKS erfordert das Zusammenspiel von verschiedenen professionsgebundenen Expertisen und somit ein besonders hohes Niveau in der multiprofessionellen und interdisziplinären Zusammenarbeit. Um einen strukturierten Versorgungsprozess zu gewährleisten, soll daher ein klinikadaptierter, standardisierter Basisalgorithmus vorhanden sein [15]. Dieser Algorithmus soll die beiden verschiedenen Vorstellungswege der Patient:innen in der ZNA beachten. Zum einen können diese durch ein Rettungsmittel (KTW, RTW, ITW, RTH) in die Notaufnahmeabteilung verbracht werden, zum anderen können sich die Patient:innen selbstständig in der Notaufnahmeabteilung vorstellen. Die initialen Vorgehensweisen unterscheiden sich hierbei, während die Versorgung im Schockraum sich gleicht (siehe Online-Zusatzmaterial Anhang 1). Je nach Zustand der Patient:innen und der allgemeinen Situation müssen die Handlungsabläufe evaluiert und situativ angepasst werden. Diese Abläufe sollen allen Beteiligten bekannt und als Merkhilfe verfügbar sein. Die initiale klinische Erstversorgung von Patient:innen mit kardiogenem Schock soll mit mindestens zwei Pflegefachkräften erfolgen [22]. Aufgrund der hohen Komplexität in der Versorgung von Patient:innen mit (infarktbedingtem) kardiogenen Schock sollte mindestens eine Pflegekraft über eine abgeschlossene Weiterbildung Notfallpflege verfügen [4].

### Empfehlung 15

- In der ZNA *soll* ein standardisiertes Anmelde- und Alarmierungsprotokoll zum Vorgehen bei unangekündigten oder vorangemeldeten Patient:innen mit (infarktbedingtem) kardiogenen Schock vorhanden sein.
- Bei Verdacht auf einen (infarktbedingten) kardiogenen Schock *soll* die höchste Dringlichkeitsstufe ausgewählt werden, welche einen sofortigen Kontakt mit dem ärztlichen Dienst erfordert. Weiterhin *soll* das Schockraumteam anhand eines internen Alarmierungsprotokolls alarmiert werden.
- Es *soll* ein klinikadaptierter, standardisierter Basisalgorithmus zur Versorgung von Patient:innen mit IKS vorhanden sein.
- Die initiale klinische Erstversorgung von Patient:innen mit kardiogenem Schock *soll* mit mindestens zwei Pflegefachkräften erfolgen.

**Starker Konsens – GoR ↑↑↑**.

Konsentiert: 7/7 der Delegierten.

### Schockraummanagement

Der Schockraum in der ZNA soll zu jeder Zeit einsatzbereit sein und eine Behandlung von kritisch kranken Menschen mit IKS ermöglichen. Hierzu müssen die Einsatzfähigkeit und Verfügbarkeit der Geräte inkl. eines 12-Kanal-EKG-Geräts und eines Echokardiographiegeräts bzw. weitere Hilfsmittel (BGA-Gerät in der Nähe, POCT-Geräte) anhand von Routineprotokollen täglich und nach jeder Nutzung durch die Pflegefachkräfte überprüft werden. Weiterhin müssen die erforderlichen Notfallmedikamente und die persönliche Schutzausrüstung ausreichend vorhanden sein. Im Fall einer Voranmeldung kann bereits ein „Fall“ im Dokumentationssystem angelegt werden.

#### Empfehlung 16

- Die Aufklärung der Patient:innen über das Vorgehen und den Ablauf im Schockraum *soll* generell durch den ärztlichen Dienst erfolgen und kann vorab und informell von der Pflegefachkraft übernommen werden.
- Um eine mögliche Schocksymptomatik zeitnah zu erkennen und um adäquat auf eine solche reagieren zu können, *soll* die Ersteinschätzung und die Weiterversorgung in der Notaufnahme von Pflegefachkräften durchgeführt werden, die in der Beurteilung klinischer Symptomatik und in der Reaktion auf diese geschult sind.
- Bereits im Schockraum *soll* eine quantitative Schmerz- und Angstanamnese anhand einer numerischen Rating-Skala oder visuellen Analogskala durchgeführt und geeignete Maßnahmen zur Schmerz- und Angstreduktion ergriffen werden.
- Eine Intervention im Herzkatheterlabor *soll* bei Patient:innen mit IKS schnellstmöglich angestrebt werden.
- Ab einer Schmerzstärke von > 3 *soll* ein schnellwirksames Analgetikum nach ärztlicher Anordnung durch die Pflegefachkraft verabreicht werden.
- Falls ein Transport in das Herzkatheterlabor nicht unverzüglich möglich ist, *sollte* bereits in der ZNA eine kontinuierliche invasive Blutdruckmessung durchgeführt werden.
- Der im Haus standardmäßig zur Koronarintervention genutzte arterielle Zugang *sollte* nicht zur Anlage eines arteriellen Katheters zur invasiven Blutdruckmessung verwendet werden.

**Starker Konsens – GoR ↑↑↑**.

Konsentiert: 7/7 der Delegierten.

### Reperfusion und Reperfusionstherapie

**Figure 1:**
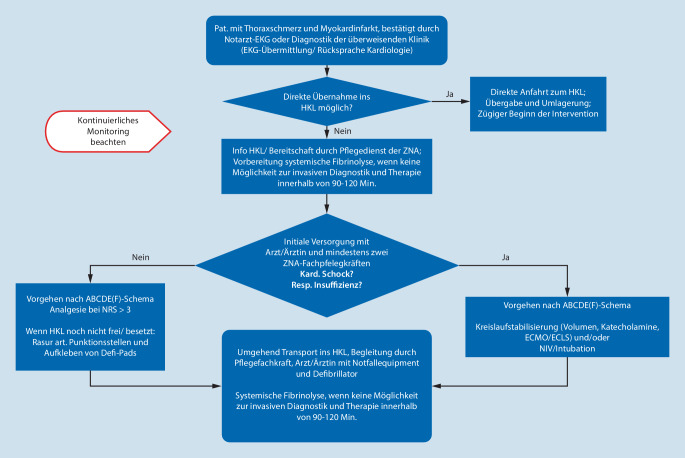

Um den Verlust von Myokardgewebe möglichst zu vermeiden oder zu minimieren, ist das elementare Ziel der Versorgung von Patient:innen mit (infarktbedingtem) kardiogenen Schock eine möglichst kurze Dauer von der Aufnahme in die Klinik bis zur Infarktintervention und daraus resultierender myokardialer Reperfusion („time is muscle“; Abb. 1 und 2).

**Figure 2:**
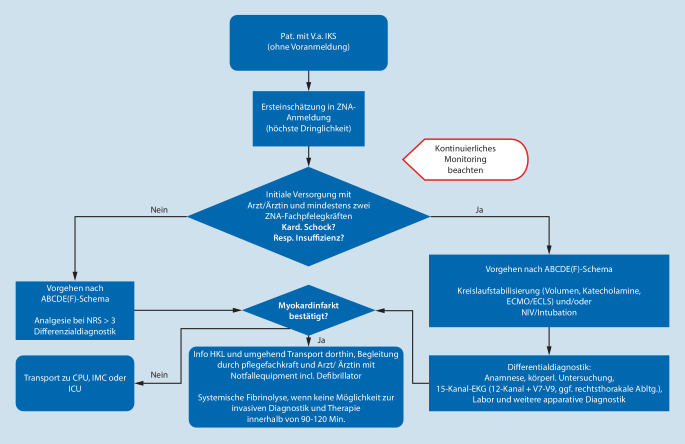

Außerhalb des Regelbetriebs soll immer mindestens ein Herzkatheter-Messplatz vorbereitet sein. Dazu gehört u. a. eine Kontrastmittelpumpe und ein Tisch mit notwendigem Sterilgut (klinische Erfahrung der Arbeitsgruppe) sowie Lagerungs- und Hilfsmittel [29].

#### Empfehlung 17

- Beim Einsatz von mechanischer Kreislaufunterstützung *soll* zusätzliches Pflegefachpersonal hinzugezogen werden (ZNA/CPU/ITS/HKL-Rufbereitschaft), das im Umgang mit diesen therapeutischen Verfahren speziell geschult und erfahren ist.
- Der Transfer vom Kathetertisch ins Bett *soll* unter kontinuierlichem Monitoring erfolgen.
- Die Aufnahme der Patient:innen in der weiterversorgenden Abteilung *soll* unter kontinuierlichem Monitoring erfolgen.
- Besteht der Verdacht auf einen kardiogenen Schock, *soll* umgehend der zuständige ärztliche Dienst und eine zweite Pflegefachkraft in die Versorgung der Patient:innen einbezogen werden.
- Therapeutisch notwendige Sofortmaßnahmen, wie NIV-Therapie, Katecholamingabe oder Reanimationsmaßnahmen *sollen* von Pflegefachkräften selbstständig eingeleitet werden.
- Zur Vorbereitung auf eine Herzkatheterintervention *können* Patient:innen mit IKS potenzielle Punktionsstellen und ggf. die Brust enthaart, Defi-Pads aufgeklebt und ein transurethraler Blasenkatheter angelegt werden.

**Starker Konsens – GoR ↑↑↑**.

Konsentiert: 7/7 der Delegierten.

Neben dem zuständigen ärztlichen Dienst sollen Patient:innentransporte von einer Pflegefachkraft begleitet werden, die in den notwendigen Handlungsabläufen geschult ist. Benötigt die:der zu transportierende Patient:in mechanische Kreislaufunterstützung, sollte der Transport von einer Pflegefachkraft begleitet werden, die über spezielle Kenntnisse im Umgang mit dieser Therapieform verfügt [5].

#### Empfehlung 18

- Vor dem Transport *sollen* Transportsauerstoff- und Druckluftflaschen auf ausreichenden Füllstand geprüft werden.
- Vor dem Transport *soll* die benötigte Energieversorgung der mitgeführten Gerätschaften auf ausreichende Ladung geprüft werden.
- Neben der Zeit des eigentlichen Transportes *soll* eine Sicherheitsspanne einkalkuliert werden, für die der Sauerstoff und die Batterieversorgung ebenfalls ausreichen muss.
- Vor dem Transport *sollen* die benötigten Spritzenpumpen, das Transportbeatmungsgerät, der Transportmonitor, das Transportabsauggerät und das Gerät zur mechanischen Kreislaufunterstützung auf ausreichende Akkukapazität überprüft werden.

**Starker Konsens – GoR ↑↑↑**.

Konsentiert: 7/7 der Delegierten.

## Pflegerische Versorgung des (infarktbedingten) kardiogenen Schocks auf der Intensivstation

Die pflegerische Versorgung von Patient:innen mit einem (infarktbedingten) kardiogenen Schock auf der Intensivstation ist von einer besonders hohen Komplexität geprägt und erfordert neben der speziellen intensivpflegerischen Expertise auch ein hohes Maß an interprofessioneller und interdisziplinärer Zusammenarbeit. Neben der allgemeinen intensivpflegerischen Versorgung liegt, insbesondere in der frühen Behandlungsphase, ein besonderer Fokus auf dem Temperatur- und Komplikationsmanagement sowie der hämodynamischen Unterstützung bzw. Überwachung mittels apparativer Hilfsmittel. Hierzu zählen eine differenzierte Katecholamintherapie sowie die Überwachung des Herzrhythmus und die Therapie ggf. auftretender Herzrhythmusstörungen. Hierdurch ergeben sich auch Herausforderungen im Management des Flüssigkeitshaushalts, in der Steuerung der Analgesie und Sedierung, der Ernährung, der Mobilisation, der Reduktion von Schmerz und Angst, der Vermeidung eines Delirs sowie der Vermeidung von Infektionen.

### Empfehlung 21

- Bei allen Patient:innen mit persistierendem (infarktbedingtem) kardiogenen Schock *soll* baldmöglichst das Herzzeitvolumen zur Therapiesteuerung im weiteren Verlauf mit einer validen Methode gemessen werden.
- Das Hauptaugenmerk *sollte* auf dem HZV liegen. Der einzige validierte prognoseanzeigende Parameter für den IKS ist der Cardiac Power (Output) (CP, CPO)/Cardiac Power Index (CPI)

**Starker Konsens – GoR ↑↑↑**.

Konsentiert: 7/7 der Delegierten.

### Management der Katecholamine

Die hämodynamische Stabilisierung nimmt einen zentralen Stellenwert bei der Behandlung von Patient:innen im kardiogenen Schock ein. Wichtig dabei ist eine Stabilisierung des Blutdrucks zur Aufrechterhaltung der Organperfusion. Der kontinuierliche Einsatz von Katecholaminen ist bei der Behandlung von Patient:innen im kardiogenen Schock jedoch nur unter einem engmaschigen Monitoring, inkl. arterieller Blutdruckmessung, und bei instabilen Patient:innen indiziert. Bei sog. „grenzwertig instabilen Patienten“ sollte keine Therapie mit Katecholaminen erfolgen [34].

Beim Umgang mit Katecholaminen ist äußerste Sorgfalt seitens der Pflegefachpersonen gefordert. Daher sollte die Steuerung der Katecholamintherapie auch nur von dafür ausgebildetem bzw. geschultem Personal vorgenommen werden.

Eine selbstständige und eigenverantwortliche Steuerung der Katecholamintherapie kann unter ärztlich angeordneter Zielwertvorgabe von weitergebildeten Pflegefachpersonen durchgeführt werden. Voraussetzung sind fundierte Kenntnisse in Pharmakodynamik und Pharmakokinetik inkl. dem Beherrschen von Nebenwirkungen und Einleiten adäquater Gegenmaßnahmen. Zur Verabreichung der Katecholamine ist ein ZVK zu bevorzugen [23]. Die Verwendung einer Trägerlösung/eines Flows zur schnelleren Verabreichung der Katecholamine wird nicht empfohlen. Nur im äußersten Notfall und zur Überbrückung ist die kurzzeitige Verabreichung über einen peripheren oder intraossären Zugang indiziert. Bei der Verwendung eines ZVK soll ein separates Lumen für die Verabreichung vasoaktiver Medikamente bestimmt werden. Die dauerhafte Applikation verschiedener Katecholamine über dasselbe Lumen ist möglich. Dabei sollte auf eine einheitliche Konzentration der einzelnen Medikamente geachtet werden. Eine hohe Verdünnung mit niedriger Konzentration kann die Sicherheit der Therapie und Katecholaminzufuhr erhöhen. Für die Zuordnung der ZVK-Lumina wird die Entwicklung und Umsetzung eines einheitlichen, hausinternen Standards empfohlen [13], der die unterschiedlichen Anordnungen der Lumina sowie deren Durchflussmenge berücksichtigt. Zu beachten ist jedoch, dass am Katecholaminschenkel weder andere Medikamente verabreicht werden noch eine Zuspritzmöglichkeit vorhanden sein soll, um Bolusgaben zu vermeiden [13].

Besonders beim Wechsel der Katecholaminspritzen ist die Fehler- bzw. Komplikationsrate sehr hoch. Hier kann es zu Pausen bzw. ungewünschten Bolusgaben kommen, welche massive Blutdruckschwankungen auslösen können. Daher wird ein überlappender Wechsel der Katecholaminspritzen empfohlen. Somit entsteht eine pausenfreie Katecholamintherapie, die hinsichtlich Bolusgaben und Free-flow-Phasen unproblematisch ist. Hierzu wird eine zusätzliche Spritzenpumpe mit gleichem Medikament und gleicher Konzentration im „Bypass“ eingesetzt Die Zusammenführung der Medikamente über 3‑Wege-Hähne soll dabei zugangsnah erfolgen. Die Kennzeichnung der Medikamente soll nach den Vorgaben der DIVI erfolgen [10]. Sämtliche Medikamentenleitungen sollen zusätzlich separat und zugangsnah gekennzeichnet werden [13].

#### Empfehlung 22

- Die Applikation von Katecholaminen *soll* über einen ZVK erfolgen.
- Die Katecholamine *sollen* über einen separaten Schenkel ohne Zuspritzmöglichkeit verabreicht werden.
- Der Wechsel der Katecholaminspritzen *soll* überlappend erfolgen.
- Die Verwendung eines „Flows“ durch zusätzliche Trägerlösungen *soll nicht* erfolgen.
- Für die Zuordnung der einzelnen ZVK-Lumina *sollte* ein einheitlicher hausinterner Standard entwickelt und umgesetzt werden.
- Die Kennzeichnung *soll* nach den Vorgaben der DIVI erfolgen.
- Die für die Patient:innen verantwortliche Pflegefachperson *soll* im Umgang mit Katecholaminen geschult sein.
- Auf eine engmaschige Dekubitusprophylaxe *soll* geachtet werden.
- Die eigenverantwortliche und selbstständige Steuerung der Katecholamintherapie *sollte* von weitergebildeten Pflegefachpersonen anhand ärztlich vorgegebener Zielparameter übernommen werden.

**Starker Konsens – GoR ↑↑↑**.

Konsentiert: 7/7 der Delegierten.

### Flüssigkeitshaushalt/Flüssigkeitsbilanz

Zur korrekten Erfassung der Urinausscheidungsmenge soll die Anlage eines transurethralen Blasenkatheters erfolgen, sofern nicht bereits eine andere Urinableitung besteht oder eine chronische Niereninsuffizienz mit bestehender Anurie vorliegt. Als Urinableitungssystem soll ein System mit integriertem Stundenurometer und Temperaturmessung verwendet werden.

#### Empfehlung 23

- Zur korrekten Erfassung der Urinausscheidungsmenge *soll* die Anlage eines transurethralen Blasenkatheters erfolgen.
- Die Überwachung des Elektrolyt- und Glukosehaushalts sowie die bedarfsweise intravenöse Substitution von Elektrolyten, Insulin und Glukose *soll* eigenständig durch die Pflegefachperson im Rahmen ärztlich vorgegebener Zielbereiche erfolgen.
- Es *soll* eine medikamentöse Aufweichung des Fäzes durchgeführt werden.

**Starker Konsens – GoR ↑↑↑**.

Konsentiert: 7/7 der Delegierten.

Zu den Themen Delir, Ernährung, mechanische Kreislaufunterstützung, Sauerstofftherapie und Hygiene verweisen wir zum einem auf die Langversion, aber auch auf die jeweils einschlägigen separaten Leitlinien und Empfehlungen zu diesen Themengebieten [1].

### Mobilisation

Für die Koronarangiographie gibt es verschiedene Zugangswege, welche die Mobilität der Patient:innen unterschiedlich beeinflussen. Im Fall eines IKS sollte derselbe Zugangsweg – transfemoral bzw. transradial – gewählt werden, den der in dieser Technik besonders erfahrene Untersucher auch bei Patient:innen mit akutem Koronarsyndrom (ACS) ohne Schock wählen würde [34]. Im Hinblick auf die Mobilisierbarkeit ist bemerkenswert, dass die gleichen Patient:innenensituationen von verschiedenen Berufsgruppen unterschiedlich eingeschätzt werden [17]. Zum Austausch über den aktuellen Stand von Wissenschaft und Technik eignen sich auch unabhängige Think Tanks und Netzwerke zur Wissensverbreitung, wie das dt. Netzwerk Frühmobilisation [27]. Eine befundorientierte Physiotherapie und daraus resultierende zielgerichtete physiotherapeutische Therapie kann bei der Betreuung der Patient:innen mit (infarktbedingtem) kardiogenen Schock erforderlich sein und soll mit den zuständigen Physiotherapeut:innen unter Berücksichtigung der aktuellen Lagerungsleitlinie initiiert, geplant und durchgeführt werden.

#### Empfehlung 29

- Eine Immobilisierung bzw. Bettruhe *soll* ärztlich angeordnet, zeitlich begrenzt und so kurz wie möglich sein (siehe DIVI Qualitätsindikator).
- Fachpflegende *sollen* die Mobilisierungsmaßnahmen nach individueller Nutzen-Risiko-Abwägung eigenständig einleiten und koordinieren, sofern keine Anordnung für eine Immobilisierung vorliegt.
- Frotteehandtücher und Felle *sollen nicht* für Lagerungsmaßnahmen verwendet werden.

**Starker Konsens – GoR ↑↑↑**.

Konsentiert: 7/7 der Delegierten.

### Nachsorge/Rehabilitation

Die Nachsorge von Patient:innen mit (infarktbedingtem) kardiogenen Schock fokussiert im innerklinischen Bereich in erster Linie die Vermeidung von postinterventionellen Komplikationen, wie bspw. einem Aneurysma spurium. Da auf Normalstationen, auf denen Patient:innen mit IKS nach ITS- oder IMC-Aufenthalt betreut werden, i. d. R. keine umfassende Monitorüberwachung gewährleistet werden kann, kommt der Krankenbeobachtung durch das Pflegefachpersonal hier eine besondere Bedeutung zu, um mögliche erneute Zustandsverschlechterungen frühzeitig zu detektieren. In diesem Zusammenhang können Frühwarnscores sowie die Implementierung von Critical-Care-Outreach-Teams oder andere medizinische Einsatzteams einen wesentlichen Beitrag zur Patient:innensicherheit leisten [2, 8, 14, 16, 24]. Das Pflegefachpersonal soll Anzeichen eines Schockrezidivs oder einer kardialen Dekompensation (Hypotonie, Bradykardie, Blässe, Zyanose, Kaltschweißigkeit, Tachypnoe, Dyspnoe, rasselndes Atemgeräusch, Jugularvenenstauung, Oligurie, Bewusstseinseintrübung, AZ-Verschlechterung) frühzeitig erkennen [3] und entsprechende therapeutische und diagnostische Maßnahmen einleiten (Herzbettlage, O_2_-Gabe, Info ärztlicher Dienst, 12-Kanal-EKG; klinische Erfahrung der Arbeitsgruppe).

#### Empfehlung 31

- Pflegefachpersonen *sollen* frühzeitig Warnhinweise auf eine Zustandsverschlechterung der Patient:innen wahrnehmen und entsprechende Maßnahmen einleiten.
- Zur postintensivstationären Nachsorge und zur Früherkennung von Zustandsverschlechterungen *kann* die Etablierung eines Critical-Care-Outreach-Teams oder eines anderen medizinischen Einsatzteams erwogen werden.
- Die Teams *sollen* auf Grundlage eines etablierten Frühwarnscores agieren.

**Starker Konsens – GoR ↑↑↑**.

Konsentiert: 7/7 der Delegierten.

Eine zentrale Aufgabe der stationären IKS-Nachsorge sind die Förderung der Autonomie und der Selbstpflegekompetenz sowie die Stärkung des Kohärenzgefühls der betroffenen Patient:innen [12].

Die Patient:innen sollen über die Möglichkeit einer Reha-Maßnahme informiert werden (Pflegefachpersonal, Sozialdienst, ärztlicher Dienst), in der die Alltagsbewältigung nach IKS intensiver eingeübt werden kann. Dabei sollte der stationären Reha der Vorzug vor der ambulanten Maßnahme gegeben werden [34].

In Österreich gibt es das Konzept „Herz-Mobil-Tirol“. Hierbei handelt es sich um ein umfassendes Versorgungsprogramm für Patient:innen mit schwerer Herzinsuffizienz. Ziel ist die nachhaltige Stabilisierung der Erkrankung, die Optimierung der medikamentösen Therapie, die Verbesserung der Eigenkompetenz der Patient:innen sowie damit verbunden ein sicherer Umgang mit der Erkrankung und eine bessere Lebensqualität für Betroffene und deren Angehörige. Das Versorgungsprogramm ist für die ersten 3 Monate nach der Entlassung aus dem Krankenhaus geplant. Sollten im Einzelfall 3 Monate nicht ausreichen, um die mit Netzwerkärzten und Pflegepersonen zu Beginn vereinbarten Ziele zu erreichen, ist eine Verlängerung um weitere 3 Monate möglich. Auch andere Gründe, wie z. B. das Überbrücken bis zu einer geplanten Operation, können eine Verlängerung erfordern (https://www.herzmobil-tirol.at/page.cfm?vpath=index).

#### Empfehlung 32

- Patient:innen nach (infarktbedingtem) kardiogenem Schock *sollen* im gesamten Klinikaufenthalt physiotherapeutische Maßnahmen zum Erhalt von Mobilität und Autonomie sowie zur Anleitung und Durchführung einer Atemtherapie erhalten.
- Eine Schulung zu Maßnahmen einer gesünderen Lebensführung *soll* im Verlauf des Klinikaufenthalts erfolgen.
- Die personelle Besetzung der kardiologischen Normalstation *soll* sich mindestens an den Bestimmungen der PpUGV orientieren.

**Starker Konsens – GoR ↑↑↑**.

Konsentiert: 7/7 der Delegierten.

## Strukturelle Rahmenbedingungen

Aufgrund der unterschiedlichen Anforderungen im Versorgungsprozess sind in den einzelnen Bereichen verschiedene strukturelle Rahmenbedingungen erforderlich.

### Zentrale Notaufnahme (ZNA)/Herzkatheterlabor/Chest Pain Unit/Intensivstation

#### Empfehlung 33

- Die personelle Ausstattung *soll* gewährleisten, das eine in Vollzeit tätige Notfallpflegekraft nicht mehr als 1200 Notfallpatient:innen im Jahr in der Initialbetreuung versorgen muss.
- Es *sollten* regelmäßige (mindestens 1‑mal Jahr) multiprofessionelle Schulungen/Simulationen zum nichttraumatologischen Schockraummanagement durchgeführt werden.

**Starker Konsens – GoR ↑↑↑**.

Konsentiert: 7/7 der Delegierten.

#### Empfehlung 34

- Das Herzkatheterlabor *soll* 24/7 verfügbar und einsatzbereit sein.
- Im Regeldienst *sollen* mindestens eine am Kathetertisch assistierende und eine weitere Pflegekraft während koronarer Interventionen im HKL anwesend sein.

**Starker Konsens – GoR ↑↑↑**.

Konsentiert: 7/7 der Delegierten.

#### Empfehlung 35

- Das Verhältnis Pflegefachkraft zu Patient:in *soll* auf einer Chest Pain Unit 1:4 nicht unterschreiten.
- Das Pflegepersonal der CPU *sollte* idealerweise die FWB „Chest Pain Unit“ oder „Intensivpflege & Anästhesie“ bzw. „Notfallpflege“ haben.

**Starker Konsens – GoR ↑↑↑**.

Konsentiert: 7/7 der Delegierten.

#### Empfehlung 36

- Die Strukturempfehlungen der DIVI in Bezug auf Personalausstattung und strukturelle Organisation einer Intensiv- und IMC Station *sollen* permanent eingehalten und umgesetzt werden.
- Die PpUGV *sollen* permanent eingehalten und umgesetzt werden.
- Die PpUGV *sollen* eine absolute Mindestbesetzung der direkten Patient:innenversorgung darstellen, jedoch keineswegs als Normalmaß und Regelbesetzung interpretiert werden.
- Es *sollten* externe unabhängige Gremien zur Kontrolle der Umsetzung von Personalvorgaben etabliert werden.
- Zur Verhinderung weiterer Komplikationen *sollten* die Qualitätsindikatoren der Intensivmedizin eingehalten werden.

**Starker Konsens – GoR **↑↑↑.

Konsentiert: 7/7 der Delegierten.

## Supplementary Information




