# Supplementary material for: Intensive care of patients with [infarct-related] cardiogenic shock: Abridged version of the S1 guideline
Source: Med Klin Intensivmed Notfmed. 2022 Aug 30;117(Suppl 2):25–36. [Article in German] doi: 10.1007/s00063-022-00945-1 (PMC9468128; doi:10.1007/s00063-022-00945-1)
Supplement: Supplementary file 1 [file 63_2022_945_MOESM1_ESM.pdf]

Online-Zusatzmaterial

Anhang 1 - Ablaufschema ZNA

Voranmeldung des Patienten

- Alarmierung des Schockraumteams nach internem Protokoll
- Vorbereitung des Schockraumes (Material, Geräte, Temperatur, Dokumente/ Formulare, Anlegen eines Falles)
- Anmeldeinformationen an das gesamte Team und an das HK-Labor weitergeben

Nach Eintreffen des Patienten:

- Übergabe durch den Rettungsdienst / Notarzt an das gesamte Team (vor der Umlagerung des Patienten)
- Gemeinsame schonende Umlagerung des Patienten
- Ersteinschätzung MTS rot / ESI 1

Unangekündigter Patient

Ersteinschätzung
MTS rot / ESI 1

- Alarmierung des Schockraumteams nach internem Protokoll
- Gewonnene Informationen an das gesamte Team weitergeben
- Anmeldung HK-Labor

Generelles Vorgehen nach dem ABCDE-Schema

Pflegefachkraft 1

- Aufklärung über das Vorgehen und den Ablauf im Schockraum
- Falls erforderlich Sauerstoffgabe; NIV-Therapie, Assistenz bei der Intubation
- Entkleidung des Patienten
- Anlage des kontinuierlichen Monitorings (EKG, SpO2, regelmäßige Blutdruckmessung, Atemfrequenz) und Erhebung der Vitalparameter
- Falls noch nicht geschehen, Anlage eines periphervenösen Zuganges und Blutentnahme
- Ggf. Unterstützung bei der Anlage eines externen Herzschrittmachers
- Parallel hierzu Einschätzung der Vigilanz Strukturierte Anamneseerhebung, Schmerz- / Angsteinschätzung und körperliche Untersuchung (gemeinsam mit dem Arzt)
- 12-Kanal EKG ggf. mit zusätzlicher Ableitung der Hinterwand (15-Kanal EKG) innerhalb der ersten 10 Min.
- DK-Anlage (mit Stundenmesseinrichtung und Temperatursonde)

Pflegefachkraft 2
(idealerweise unterstützt von einer administrativen Kraft)

- Administration mit Dokumentation der Ersteinschätzungskategorie
- Dokumentation der Maßnahmen und Wertsachen, Hilfsmittel
- Unterstützung der Pflegefachkraft 1
- Vorbereitung / Assistenz bei erforderlichen Interventionen (ZVK, Arterieller Katheter
- Organisation / Absprache mit anderen Abteilungen (z.B. HK-Labor, Radiologie, Transport- und Rettungsdienst)

- Weitere Patientenbeobachtung
  - Atem- und Kreislauffunktion
  - Mikrozirkulation (Durchblutung)
  - Diurese
  - Angst- und Schmerzreduktion
- Rasur der PCI-Punktionsstellen
- Defi-Pads anbringen (evtl. Rasur)
- Begleitung des Patienten zu den weiterversorgenden Abteilungen
- Ggf. Einleitung / Unterstützung bei der Induktion eines Temperatur-managements (TTM)

Anhang 2 - Flowchart ABCDE(F)-Schema

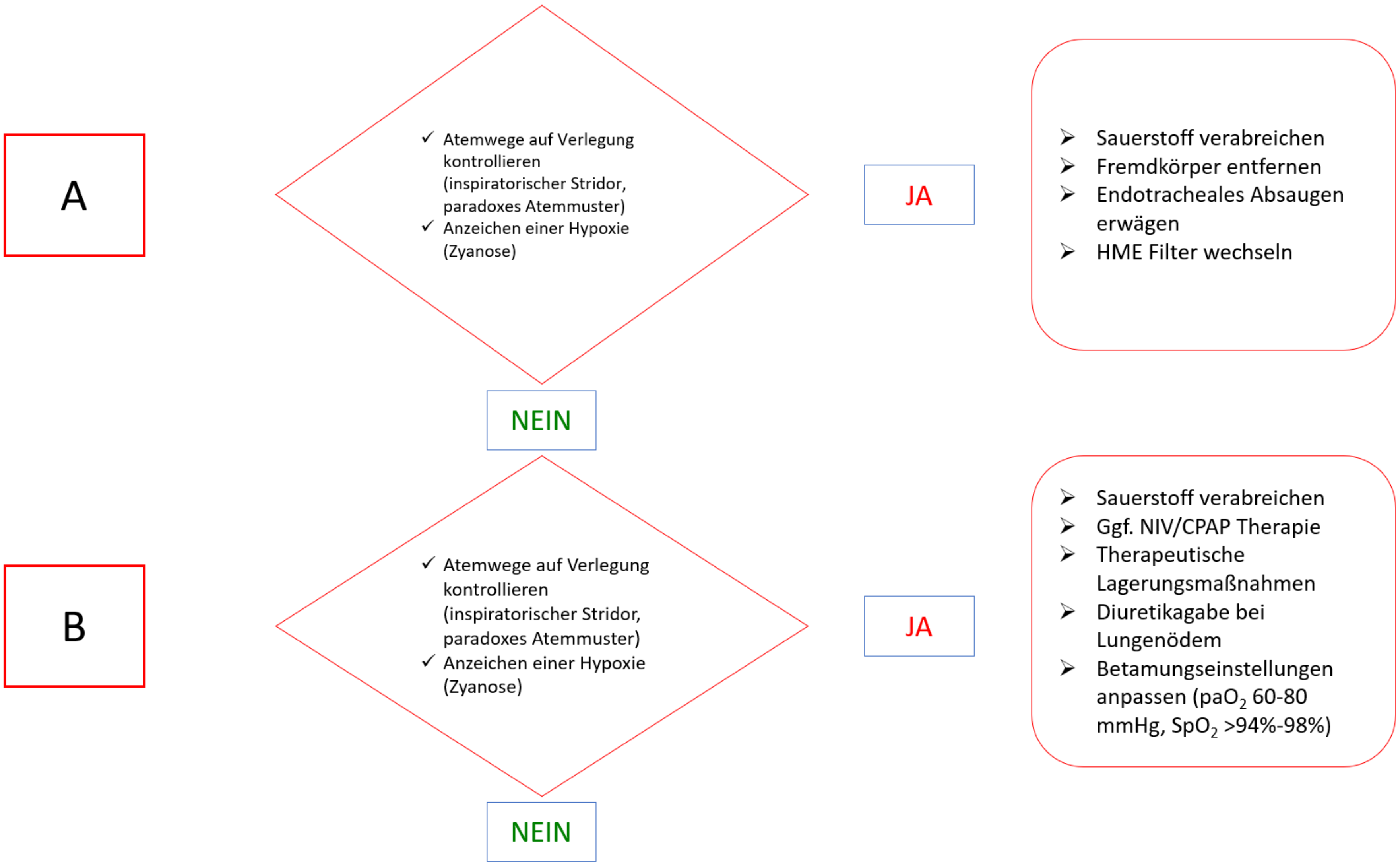

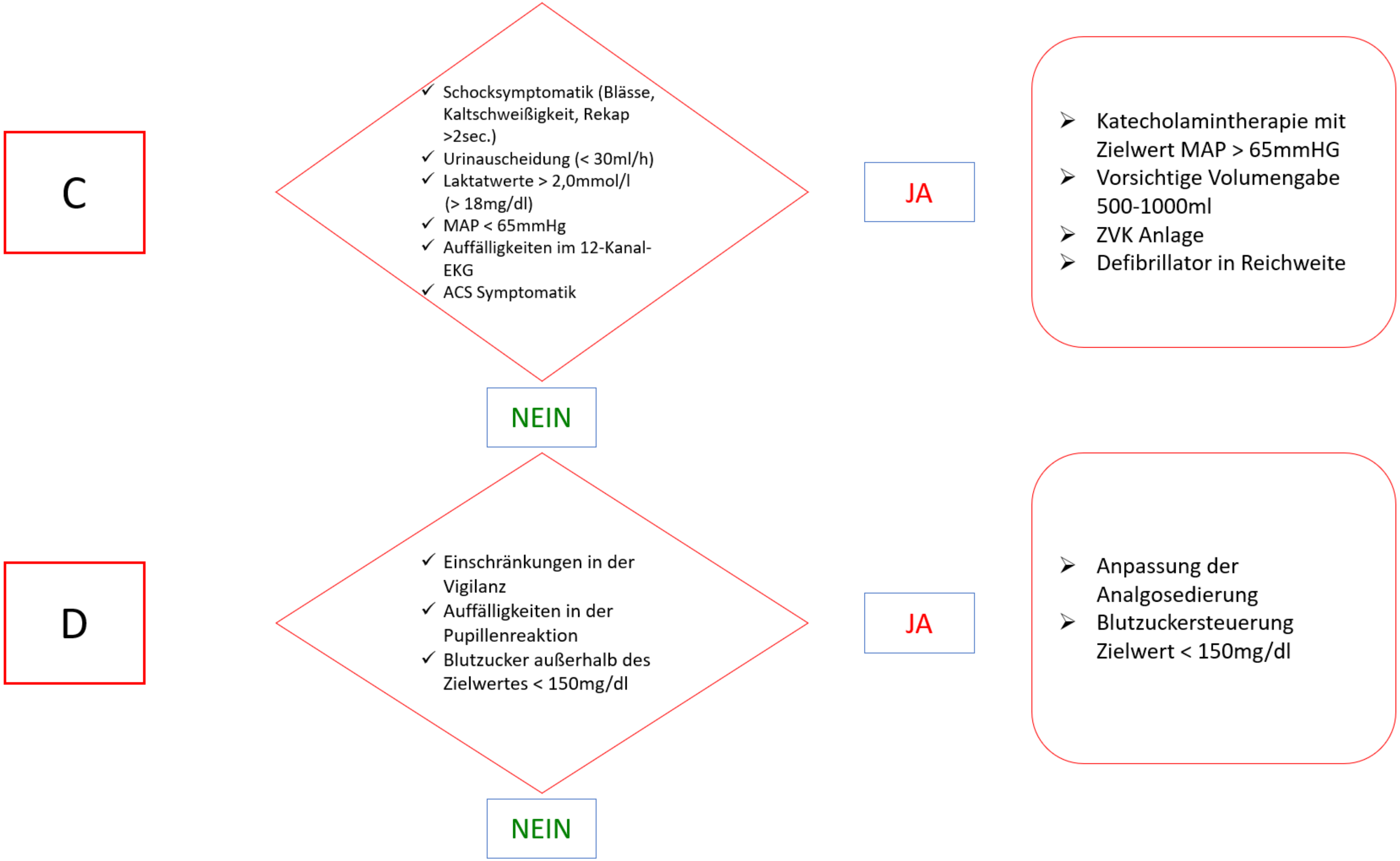

C
Schocksymptomatik (Blässe, Kaltschweißigkeit, Rekap >2sec.)
Urinauscheidung (< 30ml/h)
Laktatwerte > 2,0mmol/l (> 18mg/dl)
MAP < 65mmHg
Auffälligkeiten im 12-Kanal-EKG
ACS Symptomatik
JA
Katecholamintherapie mit Zielwert MAP > 65mmHG
Vorsichtige Volumengabe 500-1000ml
ZVK Anlage
Defibrillator in Reichweite
NEIN
D
Einschränkungen in der Vigilanz
Auffälligkeiten in der Pupillenreaktion
Blutzucker außerhalb des Zielwertes < 150mg/dl
JA
Anpassung der Analgosedierung
Blutzuckersteuerung Zielwert < 150mg/dl
NEIN

E

- ✓ Anzeichen kardialer Dekompensation (Ödeme, Marmorierung der Haut)
- ✓ Lagerung
- ✓ Temperatur

JA

- ➢ Lagerung optimieren (30° Oberkörperhoch)
- ➢ Temperaturmanagement (siehe Kapitel 4.6.)

NEIN

F

- ✓ Patientensicherheit gewährleisten – Technische Fehler ausschließen

JA

- ➢ Regelmäßige Kontrolle der apparativen Überwachungsmaßnahmen sowie der Zu- und Abgänge der Patienten

Anhang 3 - Übergabeschema SBAR

SBAR - ÜBERGABESCHEMA

SITUATION

- Name
- Alter
- Geschlecht
- Hauptdiagnose/ Aufnahmediagnose/ Eingriff/ Behandlungstag
- Besonderheiten in der Kommunikation (Schwerhörigkeit, Sehschwäche, Sprachbarriere)
- bestehende Therapielimitationen (Patientenverfügung, DNR/DNI, VaW)

BACKGROUND

- Vorerkrankungen
- Was ist akut, was chronisch? Was ist bekannt, was ist neu?
- Ernährung
- Medikation
- Soziale Situation (Angehörige, Vorsorgebevollmächtigte, Betreuer:in?)

ASSESSMENT

AIRWAY	Spontan, HFNC, NIV, intubiert, tracheotomiert (kontinuierlich/ intermittierend)
BREATHING	O_2-Bedarf, Beatmungseinstellungen, Weaningprocedere
CIRCULATION	Katecholamine, Volumenstatus, Diurese, Organunterstützung (ECLS, CRRT, andere)
DISABILITY	Delir, Orientierung, Sedierung/ Sedierungstiefe, Schmerz, BZ
EXPOSURE	Hautzustand (inkl. Wunden/ Decubitalulcera), Ernährungsstatus, Mobilisation, Pflegeprobleme

RECOMMENDATION

- Bisherige Untersuchungen und Ergbenisse
- Geplante Maßnahmen
- Tagesziele, weitere Ziele

Anhang 4 - Checkliste Antrittskontrolle

Checkliste Antrittskontrolle

Patient

- Identität prüfen
- Assessments: Vigilanz, Schmerz, Delir, Orientierung
- Auskultation Pulmo und Abdomen
- Neurologisches Assessment: Pupillenkontrolle, Sensibilität, Arm- & Beinkraft
- Fußpulse nach Katheterintervention & bei femoraler Punktion/ Kanülierung
- Inspektion der Haut & Schleimhaut (Kolorit, Turgor)
- Sichtkontrolle der Verbände/ Druckverband
- Kontrolle von ggf. Fixierungen und Bettgittern (inkl. Anordnung und Verlaufsbogen)

Bettplatz

- Kontrolle auf Vollständigkeit (interne Checkliste empfohlen)
- Kontrolle der Absaugvorrichtung auf Vollständigkeit und Funktionstüchtigkeit (Sogstärke, Absaugkatheter verschiedener Größen, Handschuhe, Mundschutz)
- Kontrolle des Bettes auf Erdung & Stromversorgung

Medikamente

- Kontrolle der Laufraten
- Kontrolle der Konzentrationen und Kompatibilitäten
- Kontrolle der Konnektion der Leitungen, Filter & Dreiwegehähne
- Richtige Zuordnung am jeweiligen Zugang nach einheitlichem hauseigenen Standard
- Kontrolle der Stromversorgung bei Spritzen- & Infusionspumpen

Monitoring

- Monitoring komplett? (EKG, NIBP/IBP, SpO_2, Temperatur)
- Hygienischer Nullabgleich invasiver Druckmessungen, auf stationseinheitliche Skalierung der Kurven achten, Transducer-Position prüfen, Druckbeutel prüfen (300mmHg)
- Kontrolle der Messintervalle, Alarmgrenzen & Alarmlautstärke
- Kontrolle erweitertes Monitoring (PiCCO, PAK, NIRS)
- BGA (art./ven.) zur Kontrolle des respiratorischen und metabolischen Verlaufs

CHECKLISTE ANTRITTSKONTROLLE

GERÄTE

Bei allen verwendeten Geräten ist auf die Sicherstellung der kontinuierlichen Energiezufuhr und die Erdung zu achten. Alle Geräte, die ein Organ unterstützen/ersetzen, müssen zwingend an eine unterbrechungsfreie Notstromversorgung angeschlossen sein. Zudem muss im Rahmen der Antrittskontrolle das Vorhandensein des notwendigen Notfallequipments für die laufenden Therapieverfahren überprüft werden.

Beatmung	→	Modus, Einstellungen, Alarmgrenzen, Befeuchtung, Handbeatmungsbeutel vorhanden?, Tubuslage, Cuffdruckkontrolle, bei TK: Ersatzkanülen/ Trachealspreizer
CRRT	→	Therapieeinstellungen, Alarmgrenzen, Dosierung der Antikoagulation, Sichtkontrolle der blutführenden Leitungen und des Filters, korrekte und zugfreie Fixierung, Einsatzdauer
ECLS	→	Therapieeinstellungen, Alarmgrenzen, Vollständigkeit Notfallequipment, Sichtkontrolle der blutführenden Leitungen, der Pumpe und des Oxygenators, Flushen des Oxygenators, Kontrolle Oberschenkelumfang/ periphere Durchblutung, korrekte & zugfreie Fixierung, ggf. pre/post - Oxygenator-BGA
Impella	→	Kontrolle P-Level, Platzierungssignal & Motorstromkurve, korrekte Konzentration der Purge - Lösung, feste Verriegelung des Tuohy-Borst-Ventils, periphere Durchblutung, korrekte & zugfreie Fixierung
Herzschrittmacher	→	Kontrolle Modus, Frequenz, feste Verriegelung des Tuohy-Borst-Ventils, korrekte & zugfreie Fixierung, Pacer-Erkennung in Monitor aktiviert

ZU- & ABLEITUNGEN

- Prüfung auf korrekte Lage und Sichtkontrolle der Eintrittsstellen & Verbände
- Gewährleistung von Zugfreiheit bei den Leitungen
- Kontrolle und Beurteilung der Diurese, ggf. Stuhlgang
- Kontrolle von Sogeinstellungen und Sekretmenge/ Beschaffenheit bei Drainagen
- Lage- & Refluxkontrolle bei einliegender Magensonde, Art & Laufrate der Sondennahrung

Anhang 5 - Checkliste ECLS

CHECKLISTE ECLS

PATIENT

- ☐ Pupillenreaktion initial stündlich, nach 24h 2-stdl.
- ☐ Lagerung des Kopfes
- ☐ Kontrolle der Kanülenfixierung
- ☐ Verbandskontrolle
- ☐ mind. 2-stdl. Dokumentation der NIRS - Parameter
- ☐ Aussehen des Beines / Umfangsdifferenz / Sensorik / Motorik mind. 8-stdl.
- ☐ Kontrolle der Fußpulse initial stündlich, nach 24h 4-stdl.
- ☐ Erfassung von Vigilanz, Schmerz, Delir mind. 8-stdl.

GERÄT

- ☐ Sichtkontrolle des gesamten Schlauchsystems auf Abknickungen, Beschädigungen, Thrombenbildung, korrekte Konnektion, Farbdifferenz
- ☐ Kontrolle der korrekten Lage und zugfreien Fixierung
- ☐ Sichtkontrolle des Oxygenators auf Thrombenbildung mittels Taschenlampe
- ☐ Oxygenator flushen für max. 10 sec.
- ☐ Oxygenator unter Patientenniveau positionieren
- ☐ Kontrolle der Geräteparameter (Umdrehungszahl/Blutfluss, Systemdrücke, SvO_2)
- ☐ Kontrolle des Gasblenders (Gasfluss, FiO_2)
- ☐ Kontrolle der Alarmgrenzen und Alarmlautstärke
- ☐ Kontrolle des Wärmetauschers (Wasserstand, zirkulierendes Rädchen)
- ☐ Notantrieb (Handkurbel) am Gerät
- ☐ Sicherer Stand des Gerätes – Bremsen festgestellt
- ☐ Stromanschluss in Notstromsteckdose
- ☐ 2 Schlauchklemmen pro Kanüle am Bett
- ☐ Notfallmedikamente nach Hausstandard vorbereitet am Bett
- ☐ EK‘s auf Abruf in Blutbank oder auf Station

Anhang 6 - Kontrollparameter ECLS-Therapie

Patientenbezogene Kontroll-Parameter der ECLS-Therapie

(Boeken et al., 2020)

Parameter	Kontrollintervall	Zielbereich
**Mittlerer arterieller Blutdruck**	kontinuierlich	>60 mmHg
**Pulskurve arteriell**	kontinuierlich	pulsatil
**Rekapillarisierungszeit**	1-8 stündlich	< 3 s
**Extremitätenperfusion (insbesondere arteriell kanülierte Extremität)**	Kontinuierlich NIRS<br>Diskontinuierlich klinisch (1x pro Schicht) + Doppler 6-stündlich	ähnlich zur nicht arteriell kanülierten Seite<br>warm, rosig, Puls nachweisbar
**Zentralvenöser Druck**	Diskontinuierlich bei Bed.	Relative Beurteilung
**Zerebrale Oxygenierung**	Kontinuierlich NIRS	Individuell verschieden, keine signifikanten Abfälle
**Diurese Stündlich**	>0.5ml/kg KG	pro Stunde
**(Zentral)venöse Sättigung**	Mind. 12stündlich	$ScvO_2 \geq 60\%$<br>$SvO_2 \geq 65\%$
**paO_2**	4-stündlich	60 – 90 mmHg
**SpO_2**	Kontinuierlich	95 – 98%
**Kapnographie**<br>**$paCO_2$**	Kontinuierlich<br>4-stündlich	Individuell nach Gap zum $paCO_2$
**pH**	4-stündlich	7,35 – 7,45
**Laktat-Plasmakonzentration**	Mind. 4-stündlich	≤2 mmol/l
**Activated Clotting Time (ACT)**	3-stündlich<br>(bei stabilen Verhältnissen 6-stündlich)	160 – 180 Sekunden
**aPTT**	4-6-stündlich bis stabil, dann 1x täglich	1.5-2x Referenzbereich
**Temperatur**	Kontinuierlich (mind. 4 - stündlich)	
**Echokardiographie**	1x täglich und bei Bed.	
**EKG**	Kontinuierlich	Keine HRST, HF >40/min und <120/min
